# Sensory Clusters of Adults With and Without Autism Spectrum Conditions

**DOI:** 10.1007/s10803-016-2976-1

**Published:** 2016-12-05

**Authors:** Marie Elwin, Agneta Schröder, Lena Ek, Tuula Wallsten, Lars Kjellin

**Affiliations:** 1 Faculty of Medicine and Health, University Health Care Research Center, Örebro University, Region Örebro County, Box 1613, 701 16 Orebro, Sweden; 2 Department of Psychology, Lund University, Lund, Sweden; 3 Centre for Clinical Research, Uppsala University, Uppsala, Sweden

**Keywords:** Autism spectrum, Adults, Sensory reactivity, Cluster analysis

## Abstract

We identified clusters of atypical sensory functioning adults with ASC by hierarchical cluster analysis. A new scale for commonly self-reported sensory reactivity was used as a measure. In a low frequency group (n = 37), all subscale scores were relatively low, in particular atypical sensory/motor reactivity. In the intermediate group (n = 17) hyperreactivity, sensory interests and sensory/motor issues were significantly elevated in relation to the first group, but not hyporeactivity. In a high frequency subgroup (n = 17) all subscale scores were significantly elevated and co-occurrence of hyper- and hyporeactivity was evident. In a population sample, a cluster of low scorers (n = 136) and high scorers relative to the other cluster (n = 26) was found. Identification of atypical sensory reactivity is important for targeting support.

## Introduction

First-hand accounts of Autism Spectrum Conditions (ASCs) regularly describe atypical sensory reactivity and perception. Intense reactions to sounds, touch, and visual stimuli are common as well as strong sensory interests (Gerland 1997; Grandin and Scariano 2005; Williams 1999). Paradoxically, sensations are often concurrently described as indistinctly perceived for example pain, temperature, or hunger (Gerland 1997; McKean 1994). Hyper- and hyporeactivity can co-occur in the same individual (Baranek et al. 2006; Elwin et al. 2012; Leekam et al. 2007). Research on sensory issues is important because atypical sensory reactivity has a major impact on daily life and affects school performance (Howe and Stagg 2016) and leisure activities (Smith and Sharp 2013). Hyperreactivity to a particular sensory stimuli can cause great distress, while multiple or enduring sensory stimuli often cause sensory overload reactions (Elwin et al. 2012; Smith and Sharp 2013). Hyporeactivity to body signals affects daily life routines (Elwin et al. 2013; Donnellan et al. 2012; Fiene and Brownlow 2015). Strong sensory interests more often have a positive impact, through development of deep interests, as exemplified by Shore (2003, p. 31).

I was fascinated with the shiny, speckled bits of quartz inside these little stones. I did this for hours on end. This fascination with the inside of stones grew into acquiring a large rock collection, which had to be lined up in perfect order, and eventually into an intense interest in geology and geography.

Sensory features were previously conceptualised as associated with but not directly diagnostic of ASC. This was changed in the new version of the Statistical Manual of Mental Disorders fifth edition (DSM-5; APA 2013). Research on sensory reactivity has focused on assessing the percentage of people with ASC that have sensory problems, and on analysing group differences between ASC groups and comparison groups, mostly non-clinical samples. A prevalence of between 69% (Baranek et al. 2006) to ~95% (Leekam et al. 2007; Tomchek and Dunn 2007) of unusual sensory reactivity in children with ASC has been reported. In comparison with non-clinical samples significant differences were found and when compared to clinical groups to a lesser (Baranek et al. 2006), or a much lesser degree (Grapel et al. 2015).

Study results on age differences in sensory reactivity in ASC are inconsistent, with indications of both decreasing unusual sensory reactivity with age (Kern et al. 2006) and increasing sensory reactivity with age (Liss et al. 2006). However, the overall picture is that sensory symptoms are still prominent in adult age (Billstedt et al. 2007; Leekam et al. 2007). It is hard to find information on sex differences of unusual sensory reactivity in ASC. Even large studies e.g. Tomcheck and Dunn (2007), or Leekam et al. (2007) do not account for sex differences. Some studies found a difference in the general population as well as in ASC, with women being more hyperreactive (Tavassoli et al. 2014) as well as having more overall sensory symptoms both in ASC and in a non-psychiatric control group (Eriksson et al. 2013).

The most common instruments used in research for measuring sensory differences are the Sensory Profile (SP; Dunn 1999) and the Adolescent Adult Sensory Profile (AASP; Brown and Dunn 2002). The theoretical basis for these scales is a general model for sensory processing applicable to all people (Dunn 1997). There are also ASC specific parent-report instruments such as the Sensory Experiences Questionnaire (SEQ; Baranek et al. 2006) with items derived after review of the literature on atypical sensory reactivity in children with ASC diagnoses including empirical studies, parental report studies, clinical reports, and conceptual models of sensory processing. The SEQ largely reflects hyper- and hypo-reactivity. Additionally, the occurrence of atypical sensory reactivity in a social or non-social context is considered in the SEQ. In contrast the instrument used in this study, the newly developed Sensory Reactivity in Autism Spectrum (SR-AS; Elwin et al. 2016), is based solely on self-reporting from adults who themselves have an ASC diagnosis and consequently their own experiences of sensory differences.

It is hard to capture the nature of sensory phenomena. There is substantial variation in sensory reactivity both between individuals with ASC (Crane et al. 2009; Leekam et al. 2007) and within individuals with ASC (Baranek et al. 2006). For example hyper- and hyporeactivity can co-occur and there can be variations due to the emotional state of the person (Smith and Sharp 2013). One way to investigate this variability is to identify clusters of individuals with similar reactivity. This has been the aim of several studies that identified sensory clusters in children and adolescents with ASC. Previous cluster analyses were conducted on parent/caregiver data (Ben-Sasson et al. 2008; Lane et al. 2014; Uljarević et al. 2016). The sensory variables entered into the analyses differ between the studies. Ausderau et al. used four sensory subscales: HYPO, HYPER, SIRS (sensory interests, repetitions, seeking) and EP (enhanced perception), in a latent profile transition analysis of a very large national sample of children with ASC aged 2–12 years. Ben-Sasson et al. (2008) used three sensory subscales: under-responsivity, over-responsivity, and sensation seeking, the participants were parents of children with ASC aged 18–33 months. Lane et al. (2014) used seven sensory channels: tactile, taste/smell, movement, visual/auditory sensitivity, underresponsive/seeks, auditory filtering, and low energy weak, in a model based cluster analysis and participants were parents of children with ASC aged 2–10 years. Uljarević et al. (2016) used the same input variables as Lane et al. (2014), but the participants differed and they included parents of children/adolescents aged 11–17 years.

Results from previous cluster analyses demonstrated an association between sensory symptoms and anxiety in children and adolescents with ASC (Uljarević et al. 2016) and between anxiety and depressive symptoms in children with ASC (Ben-Sasson et al. 2008). In a study by Pfeiffer et al. (2005) a positive correlation between anxiety and sensory defensiveness in children and adolescents with Asperger’s disorder was found as well as a significant relationship between symptoms of depression and hyporeactivity in the adolescent group. This research indicate that psychiatric comorbid symptoms and the rate of unusual sensory reactivity in children and adolescents with ASC are correlated, but we do not know if sensory symptoms are more prevalent in adult ASC with psychiatric comorbidity than in adult ASC without psychiatric comorbidity.

Cluster analyses with sensory sensitivity as input variable have been conducted in a series of studies of the general population. Aron and Aron (1997) developed the Highly Sensitive Person Scale (HSP) to measure a hypersensitive trait. In studies conducted with the HSP (2000 respondents in total) a two cluster structure was identified (Aron and Aron 1997; Aron et al. 2012). In one cluster the respondents were highly sensitive (10–35%) and in the other cluster the respondents were not highly sensitive. In light of this research we were interested in exploring the cluster structure in a sample from the general population with the SR-AS subscales as input variables.

As most studies on sensory reactivity in ASC are based on parent reports of children’s atypical sensory reactions, less certainty about sensory patterns in adults with ASC has been provided by research. While several studies have identified sensory clusters in children and adolescents referred to above, to the best of our knowledge no study to date, has studied an adult sample, using self-report and a cluster analysis approach. Sensory symptoms are described by some adults with ASC to have a strong and sometimes disruptive effect (Donnellan et al. 2012), but we do not know how these symptoms vary across the population of adults with ASC.

The main purpose of this study was to identify subgroups of adults with ASC who have similar sensory features. Based on qualitative research and former cluster analyses we hypothesized that there would be clusters of individuals with different levels of frequency of sensory symptoms. We also aimed to explore the rate of psychiatric comorbidity and possible associations between cluster membership and comorbidity in the ASC sample. Further aims were to investigate the cluster pattern for the SR-AS in a population sample and additionally possible associations between cluster membership and demographic characteristics in both samples.

## Methods

### Participants and Recruitment

Data for this study were derived from a foregoing validation study of SR-AS (Elwin et al. 2016). The ASC participants were recruited from psychiatric and habilitation services in two counties in Sweden. The inclusion period lasted from April 2012 to May 2014. Clinic-based personnel were instructed to identify and invite consecutive patients who met the inclusion criteria as they came on regular visits to the clinics. Inclusion criteria were that individuals had to be 18 years of age or older and have a clinical diagnosis of autism, Asperger disorder, or Pervasive Developmental Disorder Not Otherwise Specified (PDD-NOS; ICD-10; WHO 1992) registered in the medical records at the clinics and habilitation services involved. Further inclusion criteria, which were ensured by the personnel at the clinics and habilitation centres, were that the individuals invited to participate were able to understand the language in the questionnaire and cognitively able to answer the questions in a valid way. Their judgement was based on their personal knowledge of the patients, patient’s medical records, and prior diagnoses including intellectual level. Patients with clinical diagnoses of intellectual disability were therefore not invited. The clinic-based personnel orally informed patients eligible for participation in the study and provided an information letter. All patients were informed that their participation was voluntary and anonymous. Those who gave informed oral consent were asked to complete the SR-AS and answer background questions on gender, age, age at diagnosis, education, occupation, family circumstances, and comorbid axel I according to ICD-10. After completion the participants were asked to place the questionnaire in a prepaid envelope and seal it. The scale could be completed either at the clinic or later. In all 71 individuals with ASC diagnoses completed and returned the questionnaire.

All ASC participants were registered as patients at the psychiatric clinics and the habilitation services involved due to their ASC diagnoses or ASC diagnoses in combination with other psychiatric diagnoses. The participants had been diagnosed by multidisciplinary psychiatric teams specialising in the assessment of childhood onset neuropsychiatric conditions or by a psychiatrist and psychologist in cooperation. Global intellectual ability was always assessed with the Wechsler Intelligence Scales (WISC-III; Wechsler 1991) or the Wechsler Adult Intelligence Scale—Third Edition (WAIS-III; Wechsler 1997; WAIS-IV; Wechsler 2008). The general population participants were selected from the Swedish Population Register (SPAR 2011) which includes all residents in Sweden. A random selection was conducted of residents from the same two counties as the ASC sample. In order to to facilitate a comparison between samples the randomization was conducted with age stratified into groups reflecting the age distribution in the population with ASC who were in contact with psychiatric services included in the study. The initial population sample totalled 500. Fifteen addresses were incorrect so 485 persons received the postal questionnaire. In total 164 persons answered, thus the total response rate was 33.8%. Two questionnaires were excluded due to missing items. A letter with information about the study and the questionnaire were mailed to the sample during February 2013 with a reminder within 3 weeks. The questionnaire was identical to the one given to the ASC sample except for omission of questions about diagnoses. We did not include questions on psychiatric diagnoses in the comparison sample because it was not a volunteer sample, the participants were randomly selected from the general population and we feared that questions about diagnoses would cause non-response bias.

Both the ASC and population sample answered the questionnaire anonymously and the participants consented by filling in and sending the questionnaire. The Regional Ethical Review Board in Uppsala, Sweden, approved the study (Reg. No. 2012/049).

### Measurement

Data were collected by the SR-AS, tailored to assess sensory reactivity from the perspective of individuals with ASC. The items in the questionnaire are based on an autobiography study (Elwin et al. 2012) and an interview study (Elwin et al. 2013). The internal consistency (Cronbach’s alpha) for the total SR-AS in the combined samples was 0.96 and alphas for the subscales scores were: High awareness/Hyperreactivity 0.93, Low awareness/Hyporeactivity 0.89, strong sensory interest, 0.80, and Sensory/Motor 0.89. The validity of the scale was further explored assessing the scale’s discrimination between participants with a diagnosis of ASC from the population sample using Receiver Operating Characteristic (ROC) curve analysis and Area under the Curve (AUC). AUC was estimated at 0.93: CI 0.89–0.96, thus indicating that the probability of a randomly selected subject with ASC scoring higher than a randomly selected subject from the population was approximately 93% in this sample. The SR-AS comprises 32 items in four subscales designed to measure domains commonly reported by adults with ASC diagnoses: High awareness/Hyperreactivity (14 items; e.g. “I often feel great discomfort when other people touch me”); Low awareness/Hyporeactivity: (10 items; e.g. “I often feel no pain at times when other people think I should”); Strong sensory interests (4 items; e.g. “When I look at certain patterns or colors or hear certain sounds/tones I often find them extremely fascinating”); Sensory/Motor (4 items; e.g. “In everyday situations I often feel clumsy because I drop things, for example, or spill a lot”). The numbers of items differ in the subscales because some types of sensory reactivity like High awareness/Hyperreactivity were much more varied across senses and manifestations than, for example, the Sensory/Motor descriptions and the items are constructed to reflect the experiences described in the target group. The response format is a 4-point Likert type scale ranging from 0 (totally disagree) to 3 (totally agree). The scale scores were interpreted as follows: Totally disagree (0) = no atypical sensory reactivity, partly disagree (1) = quite low atypical sensory reactivity, partly agree (2) = quite high atypical sensory reactivity, and totally agree (3) = very high atypical sensory reactivity. The High awareness/hyper-reactivity subscale includes hyper-reactivity items and two enhanced perception items.

**Fig. 1:**
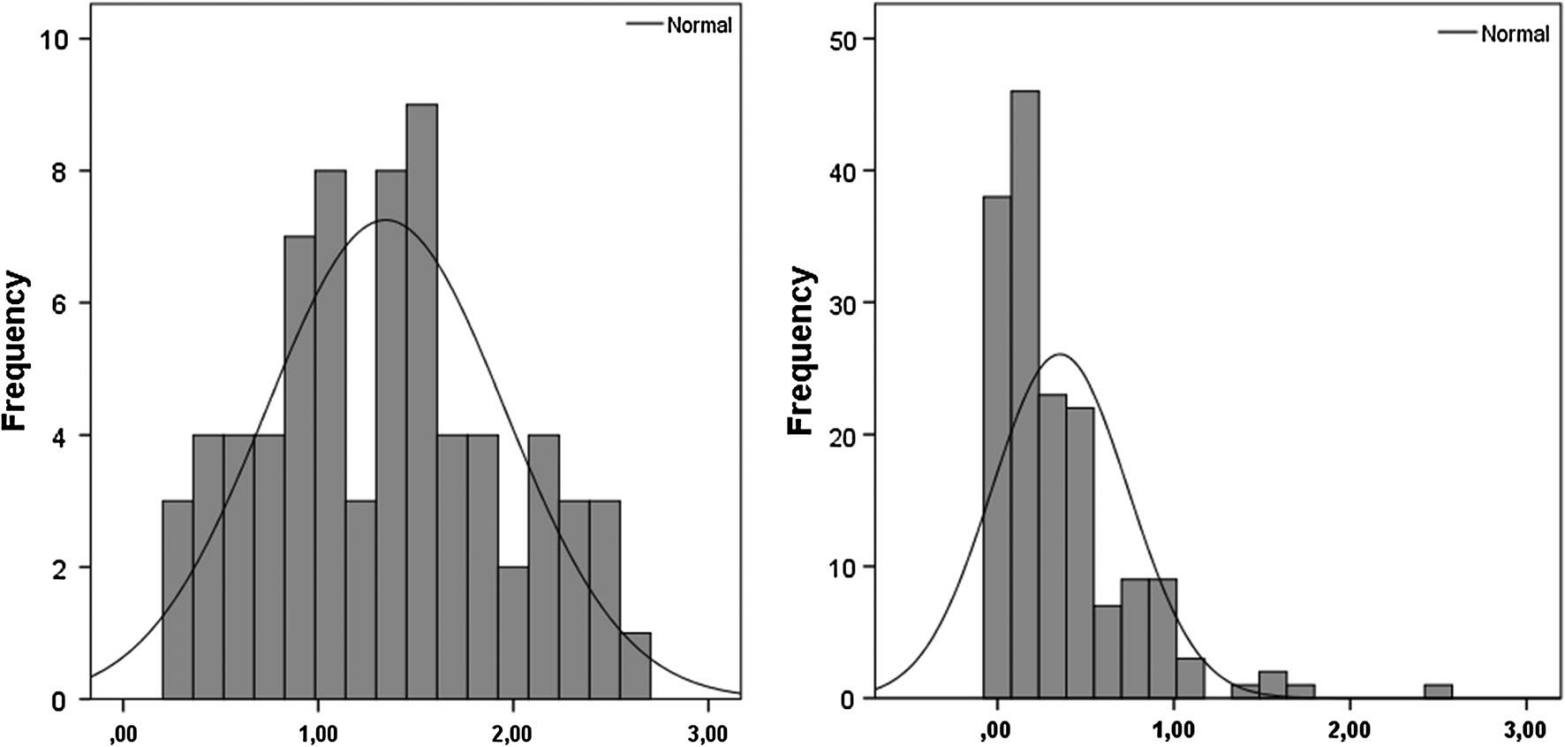
Distribution of the SR-AS mean score in the ASC and population sample

Statistics for the SR-AS in the two groups have been described earlier (Elwin et al. 2016). The scores in the ASC group had a normal distribution verified by the Kolmogorov–Smirnov test (p .20; skewness 0.2, kurtosis −0.8), whereas the population sample scores were non-normally distributed (p < .001; skewness 2.1, kurtosis 6.4) illustrated in Fig. 1.

### Statistical Analyses

The Chi square tests and Fisher’s exact test were used as appropriate to compare samples and clusters regarding demographic characteristics and comorbid diagnoses. To obtain manageable comparison group sizes, age groups, family situation, and education were allocated to three levels, and current occupation to two levels (Table 1).

**Table 1:** Demographic characteristics of participants (N = 233)

Characteristics	ASC sample n = 71 n (%)	Population sample n = 162 n (%)	*P*
Gender			.60
Women	41 (57.8)	93 (57.4)	
Men	26 (36.6)	69 (42.6)	
Missing information	4 (5.6)		
Age groups			.65
18–24	22 (31.0)	44 (27.1)	
25–44	36 (50.7)	80 (49.4)	
45–65	13 (18.3)	38 (23.5)	
Highest education			<.001*
Secondary school	21 (29.6)	11 (6.8)	
Upper-secondary school	37 (52.1)	95 (58.6)	
College/university	11 (15.5)	56 (36.6)	
Missing information	2 (2.8)		
Family situation			<.001
Married/cohabiting	19 (27)	98 (60.5)	
Single with children	8 (11)	7 (4.3)	
Single	39 (55.0)	55 (34.0)	
Missing information	5 (7.0)	2 (1.2)	
Current occupation			<.001
Working or studying	20 (28.2)	141 (87.0)	
Currently not working or studying	48 (67.6)	17 (10.5)	
Missing information	3 (4.2)	4 (2.5)	

*Pearson Chi square test, all other two-sided Fisher’s exact test

A hierarchical agglomerative cluster analysis using Ward’s method with the Euclidean distance measure was conducted (Hair et al. 1995) to identify subgroups of people with similar sensory features. Subscales obtained by previous confirmatory factor analysis (Elwin et al. 2016) were entered into the analysis. The agglomeration coefficients and dendrograms were inspected to determine the number of clusters. The stability of the hierarchical Ward’s cluster solution for the respective samples was examined using a non-hierarchical k-means cluster analysis with the number of clusters specified in advance based on the hierarchical cluster analysis solutions.

Due to the non-normal distribution of data in the population sample we used Mann–Whitney *U* test for comparison of sensory reactivity in the ASC sample in relation to the population sample and for comparison between clusters in the population sample. One way ANOVA with Tukey post hoc test was used for the comparisons of clusters in the ASC sample. Effect sizes for Mann–Whitney *U* tests were calculated (*r*) and differences in F-statistics were calculated as eta squared (proportion of variance explained by group membership). Effect sizes were evaluated in accordance with Cohen’s (1988) guidelines: a large effect for *η* ^*2*^ ≥ 0.14 and a large effect for *r* ≥ .5. A binary logistic regression analysis was performed to test which variables predict cluster membership with cluster membership dichotomized into two levels as dependent variable. The alpha level for all statistical tests was set at p < .05.

## Results

### Description and Comparison of the Samples

There were no differences in distribution by gender and age between the ASC and population samples. Almost 60% were women and around 50% belonged to the 25–44 age groups. On average the ASC sample had less advanced education and was more often single and unemployed than people in the population sample (Table 1).

A majority of the ASC participants (85%) also had self-reported a comorbid psychiatric diagnose, displayed in Table 2 ordered in ICD-10 categories.

**Table 2:** Frequency of psychiatric comorbidity according to ICD-10 classification

Comorbid psychiatric disorders	ICD-10 codes	N total
Alcohol/substance use related	F10–F19	4
Psychotic disorders	F20–29	7
Depressive disorders	F32–34	27
Bipolar	F30–31	4
Anxiety disorders	F40–F42	21
Eating disorders	F50	7
Attention-deficit/hyperactivity disorders	F90	30

More than one comorbid disorder could be reported

The total SR-AS mean score and the subscale scores were significantly higher in the ASC sample as compared to the population sample (Table 3).

**Table 3:** Mean scores (scale score 0–3) standard deviations and medians across samples

Subscale	ASC sample n = 71		Population sample n = 162		Mann–Whitney *U* test	Effect size
	M (SD)	Mdn	M (SD)	Mdn	(z) *U*	*R*
High awareness/hyper-reactivity	1.53 (0.71)	1.57	0.41 (0.43)	0.29	(−9.92) 1061.50***	−.65
Low awareness/hypo-reactivity	1.09 (0.66)	1.00	0.29 (0.40)	0.10	(−9.34) 1362.50***	−.61
Strong sensory interests	1.40 (0.73)	1.50	0.39 (0.52)	0.25	(−9.41) 1378.00***	−.62
Sensory/motor	1.26 (0.97)	1.00	0.27 (0.47)	0.00	(−8.54) 1896.50***	−.56
SR-AS total	1.35 (0.61)	1.4	0.35 (0.39)	0.22	(−10.33) 863.50***	−.68

***p < .001

### Sensory Clusters in the ASC Sample

To test the hypothesis of groups with different levels of frequency of sensory symptoms, a hierarchical cluster analysis was conducted. The agglomeration coefficients and the dendrogram generated by the cluster analysis in the ASC sample suggested a three-cluster solution (Table 4).

**Table 4:** Mean scores (standard deviations) of subscales across clusters in the ASC sample (n = 71)

Subscale	ASC cluster 1 n = 37 low	ASC cluster 2 n = 17 intermediate	ASC cluster 3 n = 17 high	ANOVA	Effect size
	M/SD	M/SD	M/SD	*F*	*η* ^*2*^
High awareness/hyperreactivity	1.15/(0.60)^a^	1.60/(0.55)^b^	2.29/(0.38)^c^	25.186***	0.43
Low awareness/hyporeactivity	0.78/(0.47)^a^	0.96/(0.45)^a^	1.91/(0.54)^b^	32.401***	0.49
Sensory interests	1.01/(0.54)^a^	1.40/(050)^b^	2.28/(0.50)^c^	32.401***	0.50
Sensory/motor	0.49/(0.39)^a^	1.81/(0.39)^b^	2.41/(0.59)^c^	105.500***	0.76

For all F statistics df is 2, 70. Clusters with different letter superscripts are significantly different by Tukey post-hoc comparisons

***p < .001

**Fig. 2:**
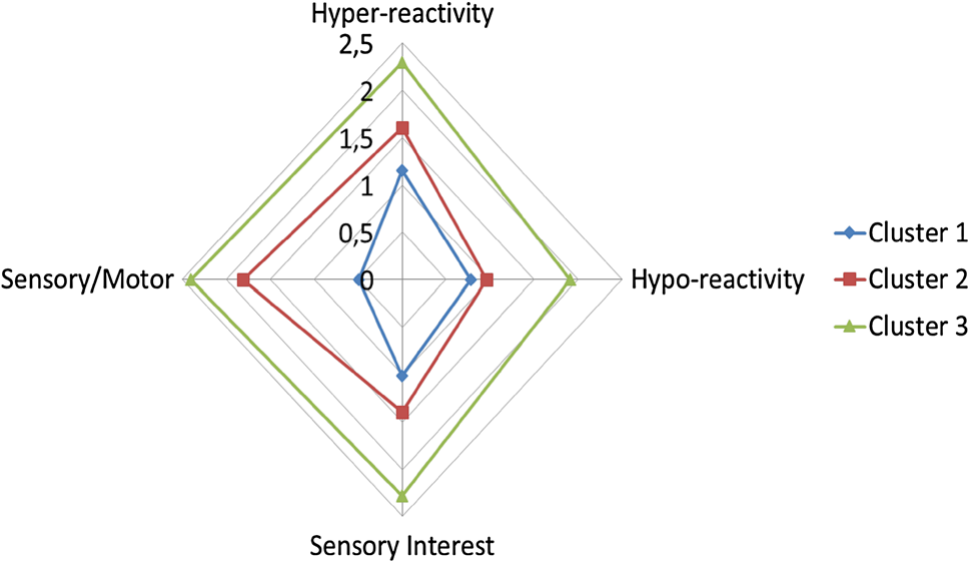
Sensory clusters of adults with autism spectrum conditions

Table 4 shows the cluster groups’ mean scores based on all individual means (scale 0–3) for the different subscales. The outcome consisted of one larger group (52%) with quite low atypical sensory reactivity and two equally sized groups (17%) with elevated scores. The differences between clusters on each sensory variable were examined with one way ANOVA. Tukey post-hoc test revealed that all subscales, except the Low awareness/Hyporeactivity subscale, differentiated significantly between all clusters. Thus Low awareness/Hyporeactivity was relatively low both in cluster one and two (Table 4). The effect sizes were large (*η* ^*2*^ = 0.43−0.76) and especially large for the Sensory/Motor subscale (Fig. 2). The three-cluster solution was validated by and had good agreement with a k-means cluster analysis and 96% of the participants in the ASC group kept their cluster membership in the k-means three-cluster solution.

There are also some relative differences between clusters as Sensory/Motor subscale in cluster one was lower (0.49) relative to the other subscales and near the population mean of 0.29. Cluster one had some atypical sensory reactivity in High awareness/Hyperreactivity, Low awareness/Hyporeactivity and Sensory interests (mean scores around 1 = quite low atypical sensory reactivity) compared to the overall means of the population sample (0.4, 0.3, and 0.4). The third cluster had elevated scores on all subscales in relation to cluster two with above quite high (2) atypical sensory reactivity on all subscales except for Low awareness/ Hyporeactivity (1.91), but this subscale was still significantly different from the subscale mean in cluster two (0.96). Cluster three represented high frequency atypical sensory reactivity on all subscales with evident concurrent High awareness/Hyperreactivity and Low awareness/ Hypo-reactivity.

### Sensory Clusters in the Population Sample

Two clusters best fitted the data in the population sample. A first large cluster of low scorers (n = 136) and a second small cluster of high scorers relative to the other cluster (n = 26; Table 5). The individuals in the second cluster had scores that deviated markedly from the subscale means in the population sample. Seven individuals had extreme values with a mean score >1.3.

**Table 5:** Mean scores (standard deviations) and medians of subscales across clusters in the population sample (n = 162)

Subscales	Cluster 1	Mdn	Cluster 2	Mdn	Mann–Whitney *U* test	Effect size
	Minimal atypical sensory reactivity n = 136		Quite low atypical sensory reactivity* n = 26			
	M (SD)		M(SD)		(z) *U*	*r*
High awareness/ hyper-reactivity	0.30 (0.30)	0.21	1.03 (0.50)	0.89	(−6.80)*** 283.50	−.53
Low awareness/hypo-reactivity	0.15 (0.16)	0.10	1.00 (0.51)	0.90	(−7.92)*** 66.50	−.62
Strong sensory interests	0.22 (0.29)	0.00	1.26 (0.55)	1.30	(−7.87)*** 130.50	−.62
Sensory/motor	0.14 (0.24)	0.00	0.96 (0.72)	1.00	(−6.64)*** 477.50	−.52

***p < .001

All factors differentiated significantly between the two clusters in the population sample (Mann–Whitney *U* test, p < .001 for all comparisons). Effect sizes were large *r* = −.52 to −.62. In the k-means cluster analysis of the population sample, 98% of the participants kept their cluster membership.

### Demographic and Clinical Characteristics of Clusters in the ASC Sample

The demographic variables age, gender, education and occupation were not associated to cluster membership in the ASC sample. We found cluster membership to be associated with the comorbid diagnoses of either ADHD or anxiety as compared to having none of these (χ^2^[1] = 5.58, *p* = .024). Alcohol/substance use diagnoses occurred only in cluster 2 and 3 (Fisher’s exact test two-sided, p = .048). There were more individuals in the first cluster (eight individuals) who did not have a comorbid diagnosis, compared to the collapsed cluster two and three (three individuals) but the difference was not significant. To investigate if ADHD or anxiety, gender or age predicts cluster membership a binary logistic regression analysis was performed with cluster membership as dependent variable dichotomized into cluster 1 as 0 and cluster 2 and 3 as 1. The total SR-AS score, sex, age group and having either ADHD or anxiety were independent variables. Alcohol/substance use included only four individuals and was not included in the analysis. The binary regression showed that the total SR-AS score was an independent predictor of cluster membership regardless of sex, age group, and ADHD and anxiety comorbidity (OR 1.16, 95% CI 1.08–1.24).

### Cluster Membership and Demographic Variables in the Population Sample

In the population sample cluster membership was associated with educational level and current occupation, whereas cluster membership was not associated with gender, age, and family situation. In the second cluster with elevated sensory reactivity the length of education was shorter compared to cluster one (elementary school 3.7% vs. 21.7%, p = .006, Fisher’s exact test two-sided) and the rates for currently not studying or working was (5.9% vs. 34.6%, P < .001, Fisher’s exact test two-sided).

## Discussion

In this study we identified sensory subgroups of adults with ASC in a psychiatric sample. The results indicated a low, intermediate, and a high atypical sensory cluster. The frequency of sensory symptoms was the main difference between clusters. The cluster solution is in line with the hypothesis of an overall frequency/severity difference between clusters (Fig. 2). In the low frequency group all measures were below the mean for the ASC sample, sensory motor reactivity in particular was low. In the intermediate group High awareness/Hyper-reactivity, Sensory interests, and Sensory/Motor issues were significantly elevated in relation to cluster one, but not Low awareness/Hyporeactivity. In the high frequency group all measures were high and co-occurrence of High awareness/Hyperreactivity and Low awareness/Hyporeactivity was evident. There seems to be considerable consistency between our results and previous cluster solutions in parent report samples. Ben-Sasson et al. (2008) used similar cluster variables (subscales) as the present study (with the exception of a sensory/motor variable in this study). They found a distinct low and high frequency subgroup and varying intermediate subgroups. Ben-Sasson et al. (2008) found low sensory seeking in the medium cluster in contrast to Ausderau et al. who found two medium clusters, one with high hyperreactivity and enhanced perception in combination with low seeking and one cluster with high hyporeactivity in combination with high sensory seeking. The reason for the discrepancies could be due to differences in age, 18–33 months in the Ben-Sasson et al. study (2008) and 2–12 years in the Ausderau et al. study (2014). The same consideration applies to the Lane et al. study (2014) ages 2–10, compared to the Uljarević et al. study (2016) ages 11–17. Input variables are the same but Lane et al. (2014) found a pattern of reactions to smell/taste and postural attentiveness in the medium clusters not found in the Uljarević et al. study (2016). Developmental level differences can be assumed to explain the differences. The results of the present study resemble the Ausderau et al. study (2014, 2016) with respect to a definite co-occurrence of elevated hyper- and hyporeactivity in a high frequency sensory subgroup alone. There is also a resemblance to the Uljarević et al. study (2016) with respect to frequency of sensory symptoms as the main discriminator between the individuals in the clusters. Other previous study results on sensory patterns in ASC are inconsistent, for example, Ermer and Dunn (1998) found a low incidence of sensory seeking, while Tomcheck and Dunn study (2007) found hyporeactivity/seeking to have the highest incidence. Uljarević et al. (2016) discuss the possibility that the relative differences in frequency between sensory reactivity types (subscales) may change with age and reconstruct into a sensory spectrum. Sensory systems are immature at birth and develop with age in typical development (Burr and Gori 2012). Sensory reactivity would differ in toddlers and young children as compared to older children, adolescents and adults, as sensory systems become increasingly refined. There is a broadening of multisensory perceptual capacity and also narrowing processes leading to increased responsiveness to stimuli in the individuals’ physical and social environment, while responsiveness to other stimuli decreases (Lewkowicz 2014). Beside developmental changes the use of compensating and coping strategies are likely to develop with age and possibly more so in individuals without intellectual disability. In qualitative research (Chamak et al. 2008; Jones et al. 2003; Robledo et al. 2012; Smith and Sharp 2013) the coping strategies used by adults with ASC are shared features of the findings. The large effect sizes of cluster group membership is another similarity between our study and findings of Ben-Sasson et al. (2008), with eta-squared and partial eta-squared ranging from 0.42 to 0.53 across studies for hyper-, hyporeactivity and sensory interests. The results from the present study and other cluster analyses indicate a sensory spectrum and thus sensory symptoms falling along a continuum. The distributions of scores in both samples are similar to the distribution of scores in ASC and comparison cases in the sensory/motor scale of the RAADS in a study by Andersen et al. (2011).

We do not know how self-report of sensory symptoms agree with parent report. There is no research comparing self-report from high functioning children/adolescents or adults with report from their parents, and we do not know if the source of information influences the results in a systematic way. Research on how well self- and parent report correlate is needed when trying to understand more about sensory reactivity and its development across the life span. For adults it is essential that their own judgements are considered. It is possible that parents are not aware of some sensory reactions, since they are not always observable, also parent’s knowledge of sensory symptoms may decrease with time. Moreover, adults with ASC and their parents may have different perspectives on sensory issues. Qualitative research on sensory reactivity cited above have shown that the many individuals with ASC place great importance to sensory stimuli and the sensory environment, and this view may not be shared by their parents. It is also possible that individuals with ASC have differences in perception that cause them not to be fully aware of their sensory reactions and both parent and self-report are needed. It is especially important to investigate the impact on the everyday lives of the group with highly elevated atypical sensory reactivity. Although sensory differences can be both positive and negative, they must nevertheless be handled by the individual. An illustration of the strong impact of sensory issues is a written comment from one of our ASC participants, who commented on an item about being fascinated by some stimuli, “Here I would need a further response step with something like: This is essentially who I am”. The self-report sale can be used as an important tool in clinical practice with adults. It provides information that can influence treatment approaches as well as make it easier for the adult patients to talk about sensory symptoms.

A surprising result is the relatively high incidence of hyporeactivity as measured by the SR-AS in the general population. In general, however, the cluster pattern for SR-AS in the population sample is similar to the cluster pattern for the highly sensitive people scale (HSP; Aron and Aron 1997, 2012). A very recent study involving children from the general population showed, in accordance with the results from our study, that approximately 12% had various types of unusual sensory reactivity (Little et al. 2016).

The rate of psychiatric comorbidity was high in this study, as is often the case in samples of psychiatrically referred adolescents and adults (Hofvander et al. 2009; Lugnegård et al. 2011). In these studies the majority of people with ASC had at least one psychiatric comorbid diagnosis, and lifetime prevalence rates reported were depressive disorders 50–77%, anxiety disorders around 50% and ADHD around 30–40%. Rates for psychotic disorders were 5–13% and eating disorders around 5%. In studies involving other types of ASC samples, the proportion of individuals with psychiatric comorbidity is smaller with a range of 20% (Hutton et al. 2008) to around 30%, experiencing severe mental health problems (Moss et al. 2015). Anxiety disorders, depressive disorders, and ADHD, are prevalent in the ASC sample in this study. For anxiety disorders the rate is ~30% approximately three times as many as the estimated ~12% population prevalence (DSM-5). For major depressive disorder (ICD-10; F32–F33) the rate was 38% in the ASC sample, five times the estimated population rate of 7% with a three times higher rate in individuals aged 18–29 years than in individuals, age 60 years or older (DSM-5). Prevalence for ADHD is 17 times higher, with 42% in this ASC sample as compared to 2.5% of adults in the general population (DSM-5).This high discrepancy to population prevalence rates for ADHD maybe due to screening for ADHD but no for other psychiatric disorders in the ASC diagnostic procedures in the clinics involved. Because the inclusion of participants in the population sample was completely at random from the population register, we think it is reasonable to assume that the prevalence of ASC in the population sample is ~1%, and that other psychiatric disorders are in the range of what is reported in DSM-5 for the general population.

The male to female ratio in this study is at odds with the sex distribution usually found in ASC of approximately 4:1 (DSM-5, APA 2013). In adult psychiatric samples for example Hofvander et al. (2009) and Eriksson et al. (2013) the sex ratio is more even. There is some evidence that females with ASC develop more concomitant psychopathology (Holtmann et al. 2007) which could explain some of the differences in male:female ratio in adult psychiatric samples.

The differences in demographic variables between the ASC and population sample were expected. Research on outcomes in ASC, recently reviewed comprehensively by Howlin (2014), has shown poor outcomes for many individuals with ASC diagnoses in education, employment, and in social or close relationships, regardless of intellectual level.

In the ASC sample a significant relationship between cluster membership and comorbidity of either anxiety or ADHD was found. A decreased regulation of response to stimulation may be related to increased mental health problems. Ben-Sasson et al. (2008) found more depression and anxiety symptoms and Uljarević et al. (2016) found more anxiety in high frequency sensory clusters.

In our study those with less education and those who were currently not working in the population sample were more represented in cluster two (people with elevated atypical sensory reactivity) indicating that this cluster may be a more troubled group. An association between health issues and higher scores on sensory measures in the general population has been found even after controlling for autistic traits (Horder et al. 2013). The lack of difference between sensory clusters on demographics in the ASC sample should be interpreted cautiously. It could be due to lack of power to detect differences in the small demographic subgroups. On the other hand very successful persons with ASC have described a broad range and high frequency of sensory issues (Elwin et al. 2012). There is also some research on the relationship between sensory symptoms and the other criteria in the second dimension of ASC. Boyd et al. (2010) found that high levels of hyperreactivity predicted high levels of repetitive behaviors, regardless of intellectual level and that seeking was significantly related to ritualistic/sameness behaviours.

There are several limitations to this study. An overall limitation is lack of more extensive validation of the SR-AS. Another major limitation is the absence of a measure of ASC traits in both samples and lack of information on psychiatric disorders, including ASC, in the population sample.

Cluster analyses results cannot be differed from the input variables (Hair et al. 1995). In our cluster analysis as in the cluster analyses by Ben-Sasson et al. (2008) input variables did not include separate sensory modalities and possible variations on sensory modality level cannot be seen in the result. Another limitation is that the ASC participants were clinically recruited and not representative for the general ASC population. Further most of the participants (85%) received their ASC diagnosis in adulthood. The participants are thought to be similar to those refereed for diagnostic evaluations in adulthood and the results from this study may not generalise to adults who were referred as young children. Moreover the comorbidity rates were high which may also limit the generalisability of the results.

## Clinical Implications and Future Directions

The need to assess atypical sensory characteristics was demonstrated. Whether or not an individual belongs to a mildly elevated or a highly elevated sensory subgroup is important information when planning support and interventions. To live with high levels of High awareness/Hyperreactivity and sensory overload cause distress. Sensations are described as a source of both pleasure and discomfort and sensory reactions in general have a stronger and sometimes disruptive impact, compared to the way they are experienced by people without autism. This is obvious in the qualitative studies referred to above. Missing items of information from the environment and from one’s own body, due to Low awareness/Hyporeactivity can also create problems in social interactions and with daily recurring routines like food, and sleep (Donnellan et al. 2012; Elwin et al. 2013; Fiene and Brownlow 2015).

There are no prior validated self-report instruments on sensory reactions tailored for adults with ASC, but even though the SR-AS offers promising validity and reliability further assessment of psychometric properties is needed. Another goal for future research on sensory reactivity in ASC is to investigate how the result from self-report compares to reports from parents. Further research also needs to focus on developmental aspects of sensory function in ASC in relation to typical development.
